# The effect of increased alcohol availability on alcohol-related health problems up to the age of 42 among children exposed *in utero*: a natural experiment

**DOI:** 10.1093/alcalc/agx069

**Published:** 2017-10-19

**Authors:** Emelie Thern, David Carslake, George Davey Smith, Per Tynelius, Finn Rasmussen

**Affiliations:** 1 Department of Public Health Sciences, Karolinska Institutet, Torsplan, Solnavägen 1E, Stockholm, Sweden; 2 MRC Integrative Epidemiology Unit, University of Bristol, Oakfield House, Oakfield Grove, Bristol, UK; 3 Population Health Sciences, Bristol Medical School, University of Bristol, Oakfield House, Oakfield Grove, Bristol, UK; 4 Centre of Epidemiology and Community Council, Health Care Services, Torsplan, Solnavägen 1E, Stockholm, Sweden; 5 Department of Health Sciences, Faculty of Medicine, Lund University, Lund, Sweden

## Abstract

**Aim:**

To examine whether exposure to increased alcohol availability *in utero* is associated with later alcohol-related health problems.

**Method:**

Register-linked population-based longitudinal study using data from a natural experiment setting, including 363 286 children born 1965–71. An experimental alcohol policy change was piloted in two regions of Sweden in 1967–68, where access to strong beer increased for 16–20 year old. Children exposed *in utero* to the policy change were compared to children born elsewhere in Sweden (excluding a border area), and to children born before and after the policy change. The outcome was obtained from the National Hospital Discharge Register using the Swedish index of alcohol-related inpatient care. Hazard ratios (HR) with 95% confidence intervals (CI) were estimated by Cox regression analysis.

**Results:**

The results suggest that children conceived by young mothers prior to the policy change but exposed to it in *utero* had a slightly increased risk of alcohol-related health problems later in life (HR 1.26, 95% CI 0.94-1.68). A tendency towards an inverse association was found among children conceived by older mothers (HR 0.88, 95% CI 0.74-1.06).

**Conclusion:**

Results obtained from a natural experiment setting found no consistent evidence of long-term health consequences among children exposed *in utero* to an alcohol policy change. Some evidence however suggested an increased risk of alcohol-related health problems among the exposed children of young mothers.

## INTRODUCTION

Evidence suggests that adolescents exposed to increased alcohol availability by a reduction in the legal minimum age for alcohol purchases are at an increased risk of binge drinking, alcohol use disorder, disability pension due to alcohol abuse and alcohol-related mortality later in life (Wagenaar and Toomey, 2002; Norberg *et al.*, 2009; Plunk *et al.*, 2013, 2016; Thern *et al.*, 2017). However, little is known about the long-term health consequences for the next generation to such an alcohol policy change.

Results from previous research on birth outcomes suggests that having a low (18 years) legal minimum age for alcohol purchases is associated with increased risk of preterm birth and fetal loss, and a higher rate of low birthweight (Fertig and Watson, 2009; Zhang and Caine, 2011; Barreca and Page, 2015; Nilsson, 2017). In the long-term, a recent Swedish study, using the same alcohol policy change as the current study, found that children of young mothers that were exposed *in utero* to a low legal minimum age for alcohol purchases (16 years) had lower educational attainment and earnings, and a higher welfare dependence at age 30 compared to the unexposed children of young mothers (Nilsson, 2017). It is therefore of interest to examine other potential long-term consequences of exposure to an alcohol policy change *in utero*, such as alcohol-related health problems.

There is considerable evidence suggesting that moderate to high prenatal alcohol exposure is associated with short and long-term effects on growth, mental development, cognition and behavioral delinquencies (Behnke *et al.*, 2013). Disagreement, however, remains regarding the effect of low levels of alcohol consumption during pregnancy (Henderson *et al.*, 2007; Flak *et al.*, 2014). There is also the question of whether there is an association between prenatal alcohol exposure and later alcohol-related health problems. The few studies that have investigated this found that moderate to high prenatal alcohol exposure was associated with an early onset of alcohol consumption and an increased risk of regular alcohol consumption, alcohol problems and alcohol disorders in early adulthood (Baer *et al.*, 1998, 2003; Alati *et al.*, 2006; Pfinder *et al.*, 2014; Cornelius *et al.*, 2015).

Sweden has a long history of restrictive alcohol policies with high age limits, high alcohol tax, strict opening hours and state-owned retail outlets (Norström and Ramstedt, 2006). The legal minimum age for purchases of strong beer was however temporarily decreased from 21 to 16 years of age in two regions of Sweden for 8.5 months during the late 1960s (SOU, 1971).

The aim of the present study is to examine whether exposure to increased alcohol availability, through this policy change, is associated with later alcohol-related health problems among the children exposed in *utero*.

## MATERIALS AND METHODS

An individual-level data set was created through record-linkage of several national registers using the Swedish unique personal identification number. The registers included were the Multi-Generation Register (MGR), the National Population and Housing Censuses (PHC), the National Hospital Discharge Register and the Cause of Death Register, kept by Statistics Sweden and the National Board of Health and Welfare.

### Description of the natural experiment

Between 1 November 1967 and 14 July 1968 an alcohol policy experiment took place in Gothenburg and Bohus County and Värmland County (referred to as the intervention area below). Within this geographical area and time period strong beer, containing 4.5–5.6% alcohol by volume, was available for purchase in regular grocery stores by individuals aged 16 years or older (SOU, 1971). Before and after the policy change, and in the regions not part of the policy experiment, strong beer could only be purchased in state-owned retail monopoly stores ‘Systembolaget’, with a legal minimum age of 21 years (SOU, 1971).

### Study population

The study population comprised all non-adopted children born in Sweden between 1 November 1965 and 15 April 1971, where their biological mother was known in the MGR (*N* = 572 293). To identify if the child was born in an intervention or control area, their biological mother's residence information was extracted from the PHC 1965, 1970 and 1975. To be included, the mother had to be registered in the intervention area or in an area unexposed to the policy change in the last PHC before the child's birth and in the first PHC after the child's birth. Children were excluded if they died before the age of 14 or if their mother emigrated from Sweden, moved between the intervention and control area or lived in a bordering area (to avoid any spill-over effect; Halland, Älvsborg, Skaraborg, Örebro and Kopparberg). The final analytical sample consisted of 363 279 children, 47 987 born in the intervention area and 315 292 born in the control area (Supplementary Figure 1).

The analytical sample was divided into four groups based on the exposure to the policy change. Cohort A included all children born before the policy change. Cohorts B and C included all children exposed to the policy change *in utero*, cohort B was conceived prior to the policy change and cohort C was conceived during the policy change. Cohort D included all children conceived after the policy change had terminated (Supplementary Table 1).

In line with previous research, the main focus was on cohort B as cohort C was more likely affected by a positive association between alcohol consumption and unplanned pregnancies (Fertig and Watson, 2009; Nilsson, 2017). Including two negative controls (cohorts A and D) that were not exposed to the strong beer policy change allows us to detect differences between the intervention and control groups which are not due to the intervention and allows for spatial pseudo-replication (Lipsitch *et al.*, 2010; Gage *et al.*, 2016). The children born before and after the policy change were seasonally matched to the children exposed to the policy change (cohorts B and C combined), as Swedish drinking habits vary seasonally with high peaks especially during summer (Norström and Ramstedt, 2006). The study was approved by the Stockholm Regional Ethical Review Board (Ref: 2016/112-31).

### Outcome measure

The outcome of alcohol-related health problems was defined as receiving inpatient care for an alcohol-related diagnosis and obtained from the National Hospital Discharge Register. Medical conditions were recorded using the Swedish version of the International Statistical Classification of Disease (ICD) version 8 (1969-1986), 9 (1987-1996) or 10 (from 1997). The Swedish index of alcohol-related inpatient care was used to define the outcome, including for example diagnoses of alcohol dependency, mental and behavioral disorders due to alcohol abuse, toxic effects of alcohol and alcoholic liver disease (Supplementary Table 2). The outcome event of interest was a person's first alcohol-related inpatient care diagnosis recorded as a principal or contributory discharge diagnosis.

### Covariates

The selection and inclusion of covariates in the analyses was based on previous research (Baer *et al.*, 2003; Alati *et al.*, 2006; Pfinder *et al.*, 2014; Nilsson, 2017). Date of birth and sex was collected from the MGR. Information on the highest level of educational attainment and socio-economic index (SEI) of either biological parent was extracted from the PHC 1970. Information on any parents’ alcohol-related health problems was extracted from the National Hospital Discharge Register and Cause of Death Register. From the PHC 1970 a regional measure of population density in the birth locality was extracted. Statistics Sweden defines a locality as an urban area with at least 200 inhabitants and where houses are no more than 200 meters apart regardless of municipal or regional boundaries (Statistics Sweden, 2012). Areas with high population density tend to have higher alcohol availability (i.e. more liquor stores and restaurants) compared to areas with lower population density (SOU, 1971; Babor *et al.*, 2010; Gruenewald, 2011; CAN, 2014; Rossow and Norström, 2013). All individual, family and regional level covariates were categorized as indicated in Table 1.

**Table 1.** Baseline characteristics of the study population, born from 1 November 1965 to 15 April 1967 (Cohort A), 1 November 1967 to 15 April 1969 (Cohorts B and C) and 1 November 1969 to 15 April 1971 (*N* = 363 279). *P*-values were estimated from Pearson's chi-square test (*χ*^2^)

	Cohort A			Cohorts B and C			Cohort D		
	Intervention area (*N* = 17 747) *n* (%)	Control area (*N* = 114 298) *n* (%)	*P*-value	Intervention area (*N* = 15 646) *n* (%)	Control area (*N* = 102 396) *n* (%)	*P*-value	Intervention area (*N* = 14 594) *n* (%)	Control area (*N* = 98 598) *N* (%)	*P*-value
Sex									
Male	9245 (52.1)	58 854 (51.5)	0.136	8017 (51.2)	52 836 (51.6)	0.402	7556 (51.8)	50 626 (51.4)	0.333
Female	8502 (47.9)	55 444 (48.5)		7629 (48.8)	49 560 (48.4)		7038 (48.2)	47 972 (48.6)	
Maternal age at conception^a^	26.0 ± 5.8	25.8 ± 5.7		26.0 ± 5.6	25.8 ± 5.4		26.0 ± 5.2	25.9 ± 5.1	
Parental SEI^b^									
High non-manual	1413 (8.0)	10 176 (8.9)	<0.001	1158 (7.4)	8937 (8.7)	<0.001	1063 (7.3)	8500 (8.6)	<0.001
Middle non-manual	3450 (19.4)	24 232 (21.2)		3117 (19.9)	21 933 (21.4)		2886 (19.8)	21 229 (21.5)	
Low non-manual	2809 (15.8)	17 757 (15.5)		2465 (15.8)	16 211 (15.8)		2422 (16.6)	15 776 (16.0)	
Self-employed/farmer	962 (5.4)	7035 (6.2)		823 (5.3)	5560 (5.4)		628 (4.3)	4282 (4.3)	
Skilled workers	6780 (38.2)	40 351 (35.3)		6065 (38.8)	36 098 (35.3)		5527 (37.9)	33 859 (34.3)	
Unskilled workers	1597 (9.0)	10 625 (9.3)		1288 (8.2)	9319 (9.1)		1177 (8.1)	9201 (9.3)	
Others, not classified	736 (4.2)	4122 (3.6)		730 (4.7)	4338 (4.3)		891 (6.1)	5751 (5.8)	
Parental level of education^b^									
Primary	7643 (43.1)	47 250 (41.3)	<0.001	6277 (40.1)	38 451 (37.6)	<0.001	5146 (35.3)	32 812 (33.3)	<0.001
Secondary	7524 (42.4)	48 380 (42.3)		7086 (45.3)	46 601 (45.5)		7175 (49.2)	47 822 (48.5)	
University and above	2300 (13.0)	17 160 (15.0)		2028 (13.0)	15 924 (15.6)		2016 (13.8)	16 455 (16.7)	
Missing	280 (1.6)	1508 (1.3)		255 (1.6)	1420 (1.4)		257 (1.8)	1509 (1.5)	
Parental alcohol-related health problems	1885 (10.6)	11 209 (9.8)	<0.001	1558 (10.0)	9587 (9.4)	0.018	1381 (9.5)	9086 (9.2)	0.335
Population density of birth locality^b,c^									
≥99 999 inhabitants	7917 (44.6)	19 324 (16.9)	<0.001	7053 (45.1)	16 662 (16.3)	<0.001	6739 (46.2)	17 844 (18.1)	<0.001
50 000–99 999 inhabitants	921 (5.2)	16 557 (14.5)		854 (5.5)	15 500 (15.1)		820 (5.6)	15 446 (15.7)	
20 000–49 999 inhabitants	1124 (6.3)	16 576 (14.5)		952 (6.1)	15 243 (14.9)		883 (6.1)	15 109 (15.3)	
10 000–19 999 inhabitants	1030 (5.8)	14 744 (12.9)		835 (5.3)	13 421 (13.1)		892 (6.1)	12 915 (13.1)	
5000–9999 inhabitants	1411 (8.0)	7071 (6.2)		1281 (8.2)	6393 (6.2)		1176 (8.1)	5827 (5.9)	
2000–4999 inhabitants	1691 (9.5)	11 264 (9.9)		1562 (10.0)	10 185 (10.0)		1395 (9.6)	9248 (9.4)	
1000–1999 inhabitants	525 (2.9)	6209 (5.4)		459 (3.0)	5466 (5.3)		407 (2.8)	4814 (4.9)	
500–999 inhabitants	512 (2.9)	4176 (3.7)		451 (2.9)	3696 (3.6)		395 (2.7)	3255 (3.3)	
200–499 inhabitants	355 (2.0)	3252 (2.9)		299 (1.9)	2822 (2.8)		238 (1.6)	2589 (2.6)	
Area not defined as a locality	2261 (12.7)	15 125 (13.2)		1900 (12.1)	13 008 (12.7)		1649 (11.3)	11 551 (11.7)	

^a^Mean and SD.

^b^Population and Housing Census 1970.

^c^A locality is defined by Statistics Sweden defined as an urban area with at least 200 inhabitants where houses are not more than 200 meters apart regardless of municipal or regional boundaries.

Cohort A: Children born prior to the policy change.

Cohort B: Children conceived prior to the change in policy and exposed to the policy change.

Cohort C: Children conceived during the policy change.

Cohort D: Children conceived after the policy terminated.

### Statistical analysis

The association between being born in the intervention area and alcohol-related health problems was estimated using Cox proportional hazards regression analysis to obtain crude and adjusted hazard ratios (HR) with 95% confidence intervals (CI). The proportional hazards assumption was verified using log-log plots and plots of Schoenfeld residuals. Follow-up began at the age of 14 and continued until the age of 42, or the date of emigration, death or first alcohol-related diagnosis, whichever came first. Follow-up ended at age 42 as this was the maximum follow-up time allowed by the registers. Additional analyses stratified by maternal age at conception (<21 and ≥21) were performed, as alcohol availability increased considerably more for individuals below the age of 21. A crude measure of maternal age at conception was calculated by subtracting nine months from maternal age at birth. In the regression analysis all individual and family level covariates (as listed in Table 1, except for population density) were included as potential confounders. In the final model, we used a stratified Cox regression model adjusting categories of population density of birth locality, allowing for a unique baseline hazard for each stratum.

Between-cohort comparisons were conducted to test if the HR obtained for cohorts B, C and D were different from those obtained for cohort A. In the Cox model, we used an interaction term between area and cohort and calculated ratio-of-ratios as linear combinations of the corresponding regression coefficients (i.e. differences of log hazards). These results were presented as HR between cohorts. Such between-cohort comparisons are more robust if the baseline incidence is fairly similar in the control groups of each cohort. To investigate this, adjusted incidences (per 10 000 person-years) were obtained as fitted values from a Poisson regression model assuming average values for all covariates included in the model (Table 2). As almost identical results were obtained in the Poisson model with the assumption of constant incidence and the adjusted Cox model, the latter model was used for the between-cohort comparisons.

**Table 2.** Descriptive statistics of alcohol-related health problems (*n* = 8417) from 1979 to 2013

	*n* cases		Crude incidence^a^		Adjusted incidence^a^	
	Intervention area	Control area	Intervention area	Control area	Intervention area	Control area
Cohort A: Total	512	2864	10.8	9.3	7.8	7.3
<21 years	128	829	12.7	12.5	8.8	9.4
≥21 years	384	2035	10.3	8.4	7.6	6.7
Cohort B: Total	191	1219	8.6	8.3	6.8	7.0
<21 years	56	289	13.5	10.2	9.5	7.6
≥21 years	135	930	7.5	7.8	6.2	7.0
Cohort C: Total	183	1079	9.3	8.5	7.4	7.2
<21 years	46	290	12.6	12.2	8.8	8.9
≥21 years	137	789	8.6	7.5	7.2	6.8
Cohort D: Total	328	2041	8.4	7.7	7.3	7.2
<21 years	79	492	12.8	11.9	8.7	8.6
≥21 years	249	1549	7.6	7.0	7.1	6.9

^a^Incidence per 10 000 person-years.

Adjusted incidence at the average values for all covariates: Adjusted for sex, month of birth (12 months) and year of birth, parents SEI (seven levels), education (three levels), and alcohol-related health problem and population density of birth locality (ten groups). Total estimates are also adjusted for maternal age at conception (continuous).

Research suggests that there is a sex difference in the vulnerability to adverse prenatal exposures (Barreca and Page, 2015; Nilsson, 2017), so an interaction between sex and increased alcohol availability in relation to the outcome of alcohol-related health problems was additionally tested. All analyses were computed using Stata Statistical Software: Release 13.

### Additional analysis

Supplementary Table 3 shows the results of an additional analysis excluding individuals with missing data on covariates (*N* = 5230). Missing values were coded as a separate category for each covariate in the main analyses, as similar results were obtained with both methods.

Individuals who moved between 1965 and 1970 to another region within the same area (intervention or control) were not excluded. As the control area is much larger than the intervention area this could introduce a bias if more mobile individuals differ in terms of factors associated with alcohol use. Thus, a sensitivity analysis was conducted excluding individuals who moved between regions but remained within the intervention/control area (*N* = 67 447). The sensitivity analysis revealed only marginally different results compared to the main analysis (Supplementary Table 4), and so individuals who moved region within the intervention or control area were retained in the main analysis.

An additional analysis was conducted with the outcome of any health problem requiring inpatient care, since fetal alcohol exposure is associated with many other health problems (Behnke *et al.*, 2013).

## RESULTS

### Baseline characteristics

The baseline characteristics of the study population, stratified by cohort, can be found in Table 1. No substantial interaction was found between sex and being born in an intervention area (*P* = 0.488), thus combined analyses for males and females are presented. Parents living in the intervention area had slightly lower educational attainment and higher alcohol-related health problems compared to parents in the control area. On a regional level, a greater proportion of individuals born in the intervention area resided in a locality with high population density compared to the individuals born in the control area.

During follow-up a total of 8417 individuals received inpatient care due to an alcohol-related diagnosis, of whom 1214 (2.5%) were born in the intervention area and 7203 (2.3%) in the control area (Table 2).

### Increased alcohol availability and alcohol-related health problems

Table 3 shows the association between being born in the intervention area and alcohol-related health problems. In the fully adjusted analysis, when considering mothers of all ages, no clear evidence of increased risk of alcohol-related health problems was found among children born in the intervention area prior to the policy change compared to same-aged children born in the control area (cohort A; HR 1.07, 95% CI 0.98, 1.18). Similar findings were found among children exposed to the policy change but conceived before (cohort B; HR 0.97, 95% CI 0.83, 1.13), among children conceived during the policy change (cohort C; HR 1.03, 95% CI 0.88, 1.21) and among children conceived after the policy changed terminated (cohort D; HR 1.02, 95% CI 0.91, 1.15).

**Table 3.** HR with 95% CIs for the association between being born in the intervention area and alcohol-related health problems and the comparison of such HR between cohorts B, C, D and A during a follow-up from the age of 14–42, stratified by maternal age at conception

	Crude HR 95% CI	Adjusted HR^a^ 95% CI	Adjusted HR^b^ 95% CI	Adjusted HR^c^ 95% CI
Cohort A: Total	1.16 (1.06, 1.28)	1.17 (1.06, 1.28)	1.12 (1.02, 1.24)	1.07 (0.98, 1.18)
<21 years	1.02 (0.84, 1.22)	1.01 (0.84, 1.22)	0.97 (0.81, 1.17)	0.93 (0.77, 1.13)
≥21 years	1.23 (1.10, 1.37)	1.22 (1.09, 1.36)	1.18 (1.06, 1.32)	1.13 (1.01, 1.26)
Cohort B: Total	1.04 (0.89, 1.21)	1.05 (0.90, 1.22)	1.01 (0.87, 1.18)	0.97 (0.83, 1.13)
<21 years	1.32 (0.99, 1.76)	1.35 (1.02, 1.80)	1.31 (0.99, 1.75)	1.26 (0.94, 1.68)
≥21 years	0.95 (0.80, 1.14)	0.95 (0.80, 1.14)	0.93 (0.77, 1.11)	0.88 (0.74, 1.06)
Cohort C: Total	1.11 (0.95, 1.30)	1.12 (0.96, 1.31)	1.08 (0.93, 1.27)	1.03 (0.88, 1.21)
<21 years	1.04 (0.76, 1.42)	1.03 (0.75, 1.40)	1.03 (0.76, 1.41)	0.99 (0.72, 1.35)
≥21 years	1.14 (0.95, 1.37)	1.14 (0.95, 1.37)	1.11 (0.92, 1.32)	1.05 (0.87, 1.26)
Cohort D: Total	1.09 (0.97, 1.23)	1.09 (0.97, 1.23)	1.07 (0.95, 1.20)	1.02 (0.91, 1.15)
<21 years	1.08 (0.86, 1.38)	1.09 (0.86, 1.38)	1.07 (0.84, 1.35)	1.02 (0.80, 1.30)
≥21 years	1.09 (0.96, 1.25)	1.09 (0.95, 1.24)	1.07 (0.93, 1.22)	1.02 (0.89, 1.17)
Cohort B vs. Cohort A: Total	0.89 (0.74, 1.06)	0.90 (0.75, 1.07)	0.90 (0.76, 1.08)	0.90 (0.76, 1.08)
<21 years	1.30 (0.93, 1.83)	1.34 (0.95, 1.88)	1.35 (0.96, 1.90)	1.35 (0.96, 1.90)
≥21 years	0.78 (0.63, 0.96)	0.78 (0.63, 0.96)	0.78 (0.63, 0.97)	0.78 (0.63, 0.96)
Cohort C vs. Cohort A: Total	0.96 (0.80, 1.15)	0.96 (0.80, 1.15)	0.97 (0.80, 1.16)	0.96 (0.80, 1.16)
<21 years	1.02 (0.71, 1.47)	1.01 (0.70, 1.46)	1.06 (0.74, 1.52)	1.06 (0.74, 1.52)
≥21 years	0.93 (0.75, 1.15)	0.94 (0.76, 1.16)	0.93 (0.75, 1.15)	0.93 (0.75, 1.15)
Cohort D vs. Cohort A: Total	0.94 (0.81, 1.09)	0.94 (0.81, 1.09)	0.95 (0.82, 1.11)	0.95 (0.82, 1.11)
<21 years	1.07 (0.79, 1.44)	1.08 (0.80, 1.45)	1.09 (0.81, 1.48)	1.09 (0.81, 1.48)
≥21 years	0.89 (0.75, 1.06)	0.90 (0.75, 1.06)	0.90 (0.76, 1.07)	0.90 (0.76, 1.07)

^a^Adjusted for sex, month of birth of the year (12 months) and year of birth. Total estimates are also adjusted for maternal age at conception (continuous).

^b^Additional adjustments for parents SEI (seven levels), education (three levels) and alcohol-related health problems.

^c^Additionally stratified by population density of birth locality (10 groups)

In the analysis stratified by maternal age, the children of young mothers in cohort B appeared to have a slightly increased risk of alcohol-related health problems if born in an intervention area (HR 1.26, 95% CI 0.94, 1.68) but this did not apply to cohort A (HR 0.93, 95% CI 0.77, 1.13), cohort C (HR 0.99, 95% CI 0.72, 1.35) or cohort D (HR 1.02, 95% CI 0.80, 1.30). Among the children of older mothers, a positive association was found in cohort A (HR 1.13, 95% CI 1.01, 1.26) and a weak inverse association was found in cohort B (HR 0.88, 95% CI 0.74, 1.06). No clear evidence of an association was found among children in cohort C (HR 1.05, 95% CI 0.87, 1.26) or cohort D (HR 1.02, 95% CI 0.89, 1.17).

### Comparisons between cohort B, C, D and A

Using cohort A as a baseline cohort, we did not find any strong evidence that the adjusted HR for cohorts B, C or D were different from cohort A when considering mothers of all ages (Table 3). In the analyses stratified by maternal age, the harmful effect of being born in an intervention area on alcohol-related health problems appears to be somewhat greater for the children of young mothers in cohort B than cohort A (relative HR 1.35, 95% CI 0.96, 1.90). The reverse was found among children of older mothers in these cohorts (relative HR 0.78, 95% CI 0.63, 0.96). The effect of being born in an intervention area in cohorts C or D did not appear to differ from cohort A, whatever the age of the mother.

### Additional analysis: all-cause health problems

During the follow-up a total of 252 440 individuals (69.5%) received inpatient care due to any health problem. In the additional analysis no strong evidence of an increased risk of general health problems was found among the children born in the intervention area, in any of the cohorts regardless the age of the mother (Supplementary Table 5).

## DISCUSSION

The results of the present study, examining the long-term health consequences of being exposed *in utero* to an alcohol policy change, did not find consistent evidence of an increased risk of alcohol-related health problems in exposed children compared to unexposed children born at the same time. However, there was some evidence suggesting an additional risk among the children of young mothers and a reduced risk among the children of older mothers.

The main aim of the policy change was to decrease the high levels of strong spirit consumption in Sweden; to achieve this goal a weaker alcohol option was made more readily available (SOU, 1971). However, by decreasing the legal minimum age for alcohol purchases the availability of alcohol drastically increased for individuals below the age of 21. The results among children conceived prior to the policy change but exposed *in utero* are in line with expectations if the policy change had an effect, as our results suggests an increased risk among children conceived by young mothers and decreased risk among children conceived by older mothers. These findings are in accordance with previous research using the same policy change to investigate its effect on several economic outcomes (Nilsson, 2017). The alcohol policy experiment was terminated earlier than planned since there were reports of increased alcohol abuse among youths (SOU, 1971). It is thus likely that young mothers’ alcohol intake was most increased by the policy change. Also, in a nationwide survey from 1968, 90% of females in Sweden reported consuming alcohol before turning 21-year old (SOU, 1971). However, the decreased risk among children conceived by older mothers is probably not due to the policy change as there was only a negligible decrease in wine and strong spirits sales in the intervention areas during the policy change (Sveriges Officiella Statistik, 1970).

The patterns among children conceived prior to the policy change but exposed *in utero* were not repeated among children conceived during the policy change. Previous research suggests that increased alcohol availability increases the risk of unintended pregnancies, preterm birth and fetal loss (Fertig and Watson, 2009; Zhang and Caine, 2011; Barreca and Page, 2015; Nilsson, 2017). As unintended pregnancies are more common among young mothers with lower educational attainment and socioeconomic status, which can affect the health of the offspring, we anticipated these children to be most vulnerable (Gipson *et al.*, 2008). However, with the present results we cannot draw any strong conclusions as to how exposure to increased alcohol availability affects the health of the children conceived by young mothers as our CIs include the null for both cohorts B and C. Further, the within-cohort analyses depend on the assumption that there are no regional differences in the outcome except those due to the intervention. As this is a strong assumption, the CIs of the within-cohort analyses should be interpreted with caution.

It is important to consider the quantity, frequency and timing of maternal alcohol consumption. Previous research using self-reported data on alcohol consumption during pregnancy found that children of mothers reporting frequent moderate or heavy episodic levels of alcohol consumption were at increased risk of alcohol use disorders in early adulthood (Baer *et al.*, 1998, 2003; Alati *et al.*, 2006; Pfinder *et al.*, 2014). Although the early stage of a pregnancy appears to be the most sensitive time period, results suggest that alcohol consumption during late pregnancy is also harmful for the offspring in relation to later alcohol-related health problems (Alati *et al.*, 2006). Since we do not have individual-level data on alcohol consumption, we can only speculate how much the alcohol policy change affected the drinking behavior of pregnant women. As the policy change took place at a time before there was general knowledge of the dangers associated with consuming alcohol while pregnant (Almond and Currie, 2011), it is assumed that pregnant women at the time consumed more alcohol than pregnant women would today.

### Strengths and limitations

Long-term follow-up, large sample size and register derived data using a natural experiment setting are all major strengths. There is less concern of confounding using a natural experiment setting such as a policy change since women who drink during pregnancy tend to differ substantially on several factors, such as nutrition, illicit drug use and other lifestyle factors, which could have an impact on the child's health (Fertig and Watson, 2009). Further, using national data are important in avoiding selection bias.

A limitation on the other hand is that we cannot draw any direct conclusion on the effect size of prenatal alcohol exposure on alcohol-related problems in middle or old age from the current results. Although the outcome of alcohol-related health problems was well defined and collected from high quality registers with full national coverage, the outcome only captures severe cases and only a small proportion of the health problems that are attributable to alcohol (Rehm *et al.*, 2010). Furthermore, inpatient care for alcohol-related diagnoses is quite rare in Sweden, thus the effect of the policy change had to be very strong in order to find an effect in the outcome (CAN, 2014). Results from our additional analysis with the outcome of any health problem requiring inpatient care, did not suggest an increased risk among the children exposed *in utero* to increased alcohol availability. Interpretation of this null results is difficult in the absence of individual-level data on alcohol consumption by pregnant woman. The analyses are based only on the inpatient care register as we were unable to include the outpatient care register due to coverage constraints. Since the 1990s there have been many changes within the medical care system in Sweden including a push for the general population to seek help at a primary healthcare center instead of a hospital. The current data would not be able to capture this time trend adequately, but it would affect the intervention and control areas equally (Anell *et al.*, 2012). Also, the children in the study might be too young to find an effect of the policy change as the majority of individuals receiving inpatient care for an alcohol-related diagnosis in Sweden are above the age of 50 (CAN, 2014). It is difficult to speculate if an effect would be found after longer follow-up, as research has identified several different underlying pathways, such as genetic, epigenetic, biological and environmental pathways, by which maternal alcohol consumption can negatively affect the outcome of the pregnancy (Baer *et al.*, 2003; Foltran *et al.*, 2011).

As the alcohol policy experiment was only conducted in two regions of Sweden we were able to include an unexposed control group born during the same time period as the exposed children. Consequently, we were able to compare exposed and unexposed children born at similar times instead of being limited to comparing children of older and younger mothers, who can differ substantially on factors related to poor infant health, such as lack of resources and poor health (Coyne and D’Onofrio, 2012). Maternal age at conception was used in the stratified analysis as opposed to maternal age at birth, in order to reduce misclassification of exposure especially among the women turning 21 during their pregnancy. Furthermore we were able to compare our estimates from the potentially exposed cohorts to a negative control cohort, which gave us the opportunity to detect any potential bias or confounding in relation to the regions in which the alcohol policy change was implemented (Lipsitch *et al.*, 2010; Gage *et al.*, 2016).

Another important factor was that medium strength beer (3.5–4.5% alcohol by volume) was introduced in grocery stores in 1965 on a national level with an age limit of 16 (Noval and Nilsson, 1984). This introduction in the early part of cohort A's gestation resulted in an increase in medium strength beer consumption, especially among the young (Noval and Nilsson, 1984), which may have affected our results.

## CONCLUSIONS

Our results obtained from a natural experiment setting suggest no consistent evidence of increased later-life alcohol-related health problems among children exposed *in utero* to an alcohol policy change. However, there was some evidence suggesting an additional risk among the exposed children of young mothers. Only a few studies have explored the effects of an alcohol policy change on the next generation, research which is important to gain a more comprehensive understanding of the potential consequences of increasing alcohol availability for the general population and especially for young adults.

## Supplementary Material

Supplementary Data
