# Supplementary material for: The effect of increased alcohol availability on alcohol-related health problems up to the age of 42 among children exposed in utero: a natural experiment
Source: Alcohol Alcohol. 2017 Oct 19;53(1):104–11. doi: 10.1093/alcalc/agx069 (PMC6019000; doi:10.1093/alcalc/agx069)
Supplement: Supplementary Data [file agx069supplemental_material_alcoalco_trackchange.docx]

**Study population**: 572 293

Born: 1 November 1965- 15 April 1971

Biological mother registered in PHC 1965, 1970, 1975^1^

Biological mother: missing information on residence

*n*= 4725 excluded

Children born in bordering counties

*n*= 98 335 excluded

Biological mother relocated between intervention and control area

*n=* 8083 excluded

**Analytic sample:** 363 279

Intervention area: 47 987

Control area: 315 292

Biological mother emigrated 1965-70/1970-75

*n*= 1570 excluded

Children not seasonally matched

*n=* 90 765 excluded

Children that died before age 14

*n=* 5536 excluded

^1^National Population and Housing Census (PHC), biological mother was registered in both PHC 1965 and 1970 if their child was born before 1970 or registered in both PHC 1970 and 1975 if their child was born after 1970.

Supplementary figure 1: Flow chart describing the selection process of the participants

| Cohort | Conceived | Born | Duration of exposure (months) | | Approximate gestational period of exposure  (+/- 0.5 months) |
| --- | --- | --- | --- | --- | --- |
|  |  |  |  |  |  |
|  |  |  | Min | Max |  |
| A | Feb-65 to July-66 | Nov-65 to Apr-67 | 0.0 | 0.0 | None |
| B | Feb-67 | Nov-67 | 0.0 | 1.0 | Months 9-9 |
| B | Mar-67 | Dec-67 | 1.0 | 2.0 | Months 8-9 |
| B | Apr-67 | Jan-68 | 2.0 | 3.0 | Months 7-9 |
| B | May-67 | Feb-68 | 3.0 | 4.0 | Months 6-9 |
| B | June-67 | Mar-68 | 4.0 | 5.0 | Months 5-9 |
| B | July-67 | Apr-68 | 5.0 | 6.0 | Months 4-9 |
| B | Aug-67 | May-68 | 6.0 | 7.0 | Months 3-9 |
| B | Sep-67 | June-68 | 7.0 | 8.0 | Months 2-9 |
| B | Oct-67 | July-68 | 8.0 | 8.5 | Months 1-9 |
| C | Nov-67 | Aug-68 | 7.5 | 8.5 | Months 0-8 |
| C | Dec-67 | Sep-68 | 6.5 | 7.5 | Months 0-7 |
| C | Jan-68 | Oct-68 | 5.5 | 6.5 | Months 0-6 |
| C | Feb-68 | Nov-68 | 4.5 | 5.5 | Months 0-5 |
| C | Mar-68 | Dec-68 | 3.5 | 4.5 | Months 0-4 |
| C | Apr-68 | Jan-69 | 2.5 | 3.5 | Months 0-3 |
| C | May-68 | Feb-69 | 1.5 | 2.5 | Months 0-2 |
| C | June-68 | Mar-69 | 0.5 | 1.5 | Months 0-1 |
| C | July-68 | Apr-69 | 0.0 | 0.5 | Months 0-0 |
| D | Feb-69 to July-70 | Nov-69 to Apr -71 | 0.0 | 0.0 | None |

Supplementary Table 1: Four cohorts based on the exposure to the policy change (1 November 1967 – 14 July 1968)

Supplementary Table 2. Swedish index of alcohol-related inpatient care and cause of death

| **ICD 8 and 9**  (1969-1986) (1987-1996) | | **ICD 10**  (from 1997) | |
| --- | --- | --- | --- |
| 291 | Alcoholic psychoses | E 24.4 | Alcohol induced pseudo-Cushing’s syndrome |
| 303 | Alcoholic dependence syndrome | F10 | Mental and behavioural disorders due to alcohol abuse |
| 305.0* | Nondependent alcohol abuse | G 31.2 | Degeneration of nervous system due to alcohol |
| 357.5* | Alcoholic polyneuropathy | G 62.1 | Alcoholic polyneuropathy |
| 425.5* | Alcoholic cardiomyopathy | G 72.1 | Alcoholic myopathy |
| 535.3* | Alcoholic gastritis | I 42.6 | Alcoholic cardiomyopathy |
| 571 | Alcoholic liver disease | K 29.2 | Alcoholic gastric |
| E860 or E980+980 | Alcohol poisoning | K70 | Alcoholic liver disease |
|  |  | K 85.2 | Alcohol-induced acute pancreatitis |
|  |  | K 86.0 | Alcohol induced chronic pancreatitis |
|  |  | R 78.0 | Alcohol in blood |
|  |  | T 51.0 | Toxic effect of alcohol |
|  |  | Y 90.0 | Evidence of alcohol involvement determined by blood alcohol level |
|  |  | Y 91.0 | Evidence of alcohol involvement determined by intoxication |
|  |  | Z71.4 | Alcohol abuse counselling and surveillance |
|  |  | Z72.1 | Alcohol use |

*Only available in ICD 9

Supplementary Table 3. Complete case analysis showing hazard ratios (HRs) with 95% confidence intervals (CIs) for the association between being born in the intervention area and alcohol-related health problems and the comparison of such HR between Cohort B, C, D and Cohort A during a follow-up from the age of 14 to 42, stratified by maternal age at conception.

|  | Crude HR  95% CI | Adjusted HR^1^  95% CI | Adjusted HR^2^  95% CI | Adjusted HR^3^  95% CI |
| --- | --- | --- | --- | --- |
| Cohort A: Total  <21 years  ≥ 21 years | 1.15 (1.04, 1.26)  0.98 (0.82, 1.18)  1.22 (1.09, 1.36) | 1.15 (1.04, 1.26)  0.97 (0.80, 1.18)  1.21 (1.08, 1.35) | 1.11 (1.01, 1.22)  0.95 (0.78, 1.15)  1.17 (1.05, 1.31) | 1.06 (0.96, 1.16)  0.91 (0.75, 1.11)  1.12 (1.00, 1.25) |
| Cohort B: Total  <21 years  ≥ 21 years | 1.03 (0.88, 1.20)  1.31 (0.98, 1.76)  0.94 (0.78, 1.13) | 1.04 (0.89, 1.21)  1.35 (1.00, 1.80)  0.94 (0.78, 1.13) | 1.01 (0.86, 1.18)  1.32 (0.98, 1.76)  0.92 (0.76, 1.10) | 0.96 (0.82, 1.12)  1.26 (0.94, 1.70)  0.87 (0.72, 1.05) |
| Cohort C: Total  <21 years  ≥ 21 years | 1.10 (0.94, 1.29)  1.01 (0.73, 1.39)  1.14 (0.95, 1.37) | 1.11 (0.95, 1.30)  1.00 (0.73, 1.37)  1.14 (0.95, 1.37) | 1.08 (0.92, 1.27)  1.00 (0.73, 1.38)  1.11 (0.92, 1.33) | 1.03 (0.87, 1.21)  0.96 (0.70, 1.32)  1.05 (0.87, 1.26) |
| Cohort D: Total  <21 years  ≥ 21 years | 1.09 (0.97, 1.23)  1.05 (0.82, 1.34)  1.11 (0.97, 1.27) | 1.09 (0.97, 1.23)  1.05 (0.82, 1.34)  1.10 (0.96, 1.26) | 1.08 (0.95, 1.21)  1.04 (0.81, 1.32)  1.09 (0.95, 1.24) | 1.03 (0.91, 1.16)  1.00 (0.79, 1.28)  1.03 (0.90, 1.18) |
| Cohort B vs. Cohort A: Total  <21 years  ≥ 21 years | 0.89 (0.75, 1.07)  1.35 (0.92, 1.91)  0.78 (0.63, 0.96) | 0.90 (0.75, 1.08)  1.38 (0.97, 1.96)  0.78 (0.63, 0.96) | 0.91 (0.76, 1.09)  1.39 (0.98, 1.97)  0.78 (0.63, 0.97) | 0.91 (0.76, 1.09)  1.39 (0.99, 1.97)  0.78 (0.63, 0.97) |
| Cohort C vs. Cohort A: Total  <21 years  ≥ 21 years | 0.96 (0.80, 1.16)  1.04 (0.71, 1.50)  0.94 (0.76, 1.16) | 0.97 (0.80, 1.17)  1.02 (0.71, 1.49)  0.94 (0.76, 1.17) | 0.98 (0.81, 1.18)  1.06 (0.73, 1.53)  0.95 (0.76, 1.17) | 0.97 (0.81, 1.17)  1.06 (0.72, 1.53)  0.94 (0.76, 1.17) |
| Cohort D vs. Cohort A: Total  <21 years  ≥ 21 years | 0.95 (0.82, 1.11)  1.07 (0.79, 1.46)  0.91 (0.77, 1.09) | 0.95 (0.82, 1.11)  1.08 (0.79, 1.48)  0.91 (0.77, 1.09) | 0.97 (0.82, 1.13)  1.09 (0.80, 1.49)  0.93 (0.78, 1.10) | 0.97 (0.83, 1.13)  1.10 (0.80, 1.50)  0.93 (0.78, 1.10) |

^*^Excluding 5230 individuals with missing data on covariates

^1^Adjusted for sex, month of birth of the year (12 months) and year of birth. Total estimates are also adjusted for maternal age at conception (continuous).

^2^Additional adjustments for parents SEI (seven levels), education (three levels) and alcohol-related health problems.

^3^Additionally stratified by population density of birth locality (ten groups)

Supplementary Table 4. Sensitivity analysis showing hazard ratios (HRs) with 95% confidence intervals (CIs) for the association between being born in the intervention area and alcohol-related health problems and the comparison of such HR between Cohort B, C, D and Cohort A during a follow-up from the age of 14 to 42, stratified by maternal age at conception.

|  | Crude HR  95% CI | Adjusted HR^1^  95% CI | Adjusted HR^2^  95% CI | Adjusted HR^3^  95% CI |
| --- | --- | --- | --- | --- |
| Cohort A: Total  <21 years  ≥ 21 years | 1.19 (1.08, 1.31)  1.04 (0.85, 1.25)  1.25 (1.12, 1.40) | 1.18 (1.07, 1.30)  1.04 (0.85, 1.25)  1.24 (1.11, 1.39) | 1.16 (1.05, 1.28)  1.00 (0.82, 1.21)  1.23 (1.10, 1.37) | 1.09 (0.99, 1.21)  0.94 (0.78, 1.15)  1.15 (1.03, 1.29) |
| Cohort B: Total  <21 years  ≥ 21 years | 1.03 (0.88, 1.20)  1.25 (0.93, 1.69)  0.96 (0.80, 1.16) | 1.03 (0.88, 1.21)  1.28 (0.96, 1.73)  0.96 (0.80, 1.15) | 1.02 (0.87, 1.20)  1.25 (0.93, 1.68)  0.95 (0.79, 1.14) | 0.96 (0.82, 1.13)  1.19 (0.88, 1.60)  0.89 (0.74, 1.07) |
| Cohort C: Total  <21 years  ≥ 21 years | 1.11 (0.94, 1.30)  1.05 (0.76, 1.45)  1.13 (0.94, 1.37) | 1.11 (0.95, 1.31)  1.04 (0.75, 1.43)  1.14 (0.94, 1.37) | 1.10 (0.93, 1.29)  1.04 (0.76, 1.44)  1.12 (0.92, 1.35) | 1.03 (0.87, 1.21)  0.99 (0.71, 1.36)  1.04 (0.86, 1.26) |
| Cohort D: Total  <21 years  ≥ 21 years | 1.13 (1.00, 1.27)  1.12 (0.87, 1.43)  1.13 (0.98, 1.30) | 1.13 (1.00, 1.28)  1.12 (0.88, 1.44)  1.12 (0.98, 1.29) | 1.11 (0.98, 1.25)  1.10 (0.86, 1.41)  1.10 (0.95, 1.27) | 1.05 (0.93, 1.19)  1.05 (0.82, 1.35)  1.05 (0.91, 1.21) |
| Cohort B vs. Cohort A: Total  <21 years  ≥ 21 years | 0.87 (0.72, 1.04)  1.21 (0.85, 1.73)  0.77 (0.61, 0.96) | 0.87 (0.73, 1.05)  1.24 (0.87, 1.77)  0.77 (0.62, 0.96) | 0.88 (0.73, 1.06)  1.26 (0.88, 1.79)  0.77 (0.62, 0.96) | 0.88 (0.73, 1.06)  1.26 (0.88, 1.79)  0.77 (0.62, 0.96) |
| Cohort C vs. Cohort A: Total  <21 years  ≥ 21 years | 0.93 (0.77, 1.13)  1.01 (0.70, 1.47)  0.91 (0.73, 1.13) | 0.94 (0.78, 1.14)  1.01 (0.69, 1.46)  0.91 (0.74, 1.14) | 0.95 (0.78, 1.14)  1.05 (0.72, 1.52)  0.91 (0.73, 1.13) | 0.94 (0.78, 1.14)  1.04 (0.72, 1.52)  0.91 (0.73, 1.13) |
| Cohort D vs. Cohort A: Total  <21 years  ≥ 21 years | 0.95 (0.82, 1.11)  1.08 (0.79, 1.48)  0.91 (0.76, 1.08) | 0.96 (0.82, 1.12)  1.09 (0.80, 1.49)  0.91 (0.76, 1.08) | 0.95 (0.82, 1.11)  1.11 (0.81, 1.51)  0.90 (0.75, 1.08) | 0.96 (0.82, 1.13)  1.11 (0.81, 1.52)  0.91 (0.75, 1.09) |

^*^Excluding 67 447 individuals who moved between regions within the intervention or control area

^1^Adjusted for sex, month of birth of the year (12 months) and year of birth. Total estimates are also adjusted for maternal age at conception (continuous).

^2^Additional adjustments for parents SEI (seven levels), education (three levels) and alcohol-related health problems.

^3^Additionally stratified by population density of birth locality (ten groups)

Supplementary Table 5. Sensitivity analysis showing hazard ratios (HRs) with 95% confidence intervals (CIs) for the association between being born in the intervention area and all-cause health problems and the comparison of such HR between Cohort B, C, D and Cohort A during a follow-up from the age of 14 to 42, stratified by maternal age at conception.

|  | Crude HR  95% CI | Adjusted HR^1^  95% CI | Adjusted HR^2^  95% CI | Adjusted HR^3^  95% CI |
| --- | --- | --- | --- | --- |
| Cohort A: Total  <21 years  ≥ 21 years | 0.97 (0.96, 0.99)  0.96 (0.93, 1.00)  0.98 (0.96, 1.00) | 0.99 (0.97, 1.00)  0.98 (0.94, 1.02)  0.98 (0.96, 1.01) | 0.98 (0.96, 0.99)  0.97 (0.93, 1.01)  0.97 (0.95, 0.99) | 0.98 (0.96, 1.00)  0.97 (0.93, 1.01)  0.98 (0.96, 1.00) |
| Cohort B: Total  <21 years  ≥ 21 years | 1.00 (0.97, 1.02)  0.98 (0.92, 1.04)  1.00 (0.97, 1.03) | 1.00 (0.97, 1.02)  0.94 (0.88, 1.00)  1.01 (0.98, 1.04) | 0.98 (0.96, 1.01)  0.93 (0.87, 0.99)  1.00 (0.97, 1.03). | 0.99 (0.97, 1.02)  0.93 (0.87, 0.99)  1.00 (0.97, 1.04) |
| Cohort C: Total  <21 years  ≥ 21 years | 0.99 (0.96, 1.01)  0.95 (0.89, 1.01)  0.99 (0.96, 1.03) | 0.99 (0.96, 1.02)  0.97 (0.90, 1.03)  0.99 (0.96, 1.02) | 0.98 (0.95, 1.01)  0.96 (0.90, 1.03)  0.98 (0.94, 1.01) | 0.98 (0.96, 1.01)  0.97 (0.90, 1.04)  0.99 (0.95, 1.02) |
| Cohort D: Total  <21 years  ≥ 21 years | 1.01 (0.99, 1.03)  0.99 (0.94, 1.05)  1.01 (0.99, 1.03) | 1.02 (1.00, 1.04)  0.98 (0.93, 1.03)  1.02 (1.00, 1.05) | 1.01 (0.99, 1.03)  0.98 (0.93, 1.03)  1.01 (0.99, 1.04) | 1.02 (0.99, 1.04)  0.98 (0.93, 1.03)  1.02 (1.00, 1.05) |
| Cohort B vs. Cohort A: Total  <21 years  ≥ 21 years | 1.02 (0.99, 1.06)  1.01 (0.94, 1.09)  1.03 (0.99, 1.07) | 1.01 (0.98, 1.05)  0.96 (0.89, 1.03)  1.02 (0.99, 1.06) | 1.01 (0.98, 1.04)  0.96 (0.89, 1.03)  1.02 (0.99, 1.06) | 1.01 (0.98, 1.04)  0.96 (0.89, 1.03)  1.02 (0.99, 1.06) |
| Cohort C vs. Cohort A: Total  <21 years  ≥ 21 years | 1.01 (0.98, 1.05)  0.98 (0.91, 1.06)  1.02 (0.98, 1.06) | 1.00 (0.97, 1.04)  0.99 (0.91, 1.07)  1.00 (0.96, 1.04) | 1.00 (0.97, 1.04)  1.00 (0.92, 1.08)  1.00 (0.96, 1.04) | 1.00 (0.97, 1.04)  1.00 (0.92, 1.08)  1.00 (0.97, 1.04) |
| Cohort D vs. Cohort A: Total  <21 years  ≥ 21 years | 1.04 (1.01, 1.07)  1.03 (0.96, 1.10)  1.04 (1.00, 1.07) | 1.03 (1.00, 1.06)  1.00 (0.94, 1.07)  1.04 (1.01, 1.07) | 1.03 (1.01, 1.06)  1.01 (0.95, 1.08)  1.04 (1.01, 1.07) | 1.03 (1.01, 1.06)  1.01 (0.94, 1.08)  1.04 (1.01, 1.07) |

^1^Adjusted for sex, month of birth of the year (12 months) and year of birth. Total estimates are also adjusted for maternal age at conception (continuous).

^2^Additional adjustments for parents SEI (seven levels), education (three levels) and alcohol-related health problems.

^3^Additionally stratified by population density of birth locality (ten groups)
